# Impaired Lysosome Reformation in Chloroquine-Treated Retinal Pigment Epithelial Cells

**DOI:** 10.1167/iovs.64.11.10

**Published:** 2023-08-07

**Authors:** M. Helena Cardoso, Michael J. Hall, Thomas Burgoyne, Pedro Fale, Tina Storm, Cristina Escrevente, Pedro Antas, Miguel C. Seabra, Clare E. Futter

**Affiliations:** 1 iNOVA4Health, NOVA Medical School, Faculdade de Ciências Médicas, Universidade NOVA de Lisboa; Lisboa, Portugal; 2 UCL Institute of Ophthalmology, London, United Kingdom

**Keywords:** chloroquine, retinal pigment epithelium, lysosome

## Abstract

**Purpose:**

To model the in vivo effects of chloroquine on the retinal pigment epithelium in experimentally tractable cell culture systems and determine the effects of mild chloroquine treatment on lysosome function and turnover.

**Methods:**

Effects of low-dose chloroquine treatment on lysosomal function and accessibility to newly endocytosed cargo were investigated in primary and embryonic stem cell–derived RPE cells and ARPE19 cells using fluorescence and electron microscopy of fluorescent and gold-labeled probes. Lysosomal protein expression and accumulation were measured by quantitative PCR and Western blotting.

**Results:**

Initial chloroquine-induced lysosome neutralization was followed by partial recovery, lysosomal expansion, and accumulation of undegraded endocytic, phagocytic, and autophagic cargo and inhibition of cathepsin D processing. Accumulation of enlarged lysosomes was accompanied by a gradual loss of accessibility of these structures to the endocytic pathway, implying impaired lysosome reformation. Chloroquine-induced accumulation of pro–cathepsin D, as well as the lysosomal membrane protein, LAMP1, was reproduced by treatment with protease inhibitors and preceded changes in lysosomal gene expression.

**Conclusions:**

Low-dose chloroquine treatment inhibits lysosome reformation, causing a gradual depletion of lysosomes able to interact with cargo-carrying vacuoles and degrade their content. The resulting accumulation of newly synthesized pro–cathepsin D and LAMP1 reflects inhibition of normal turnover of lysosomal constituents and possibly lysosomes themselves. A better understanding of the mechanisms underlying lysosome reformation may reveal new targets for the treatment of chloroquine-induced retinopathy.

Chloroquine (CQ) and hydroxychloroquine (HCQ) are well known as antimalarial drugs. They readily cross membranes at neutral pH but become protonated and trapped within acidic lysosomes, where they can elevate lysosomal pH, inhibiting lysosomal enzyme activity. CQ and HCQ are also Food and Drug Administration–licensed autophagy inhibitors, are widely used for the treatment of autoimmune disorders, and are receiving increasing attention in cancer treatment in clinical settings, where autophagy has been implicated in resistance to therapy.^1^^,^^2^ CQ and, to a lesser extent, HCQ induce retinopathy in a subset of patients, characterized by central vision loss, cone maculopathy, and bull's-eye maculopathy (loss of RPE cells in an expanding ring from the fovea).^3^ CQ-induced retinopathy thus resembles features of other retinal degenerations, including Stargardt disease and age-related macular disease (AMD). The trigger for CQ-induced retinopathy remains to be established, but impaired lysosome function within the RPE, exacerbated by environmental and genetic risk factors, is a likely contributor.

We hypothesize that the huge degradative burden of the RPE renders these cells particularly sensitive to CQ-induced partial lysosome dysfunction. The RPE lies adjacent to the photoreceptor outer segments (POSs), which are subject to extensive photo-oxidative damage so that the entire POS is replaced about every 10 days. Each day, the distal 10% of the POS is phagocytosed and degraded by the RPE.^4^^–^^6^ In aging RPE and in AMD, the lipofuscin that accumulates contains bisretinoids, indicating that it is at least in part derived from POS.^7^ Extracellular deposits (drusen) are also a hallmark of AMD, although their origin is less clear. Consistent with the idea that lysosome dysfunction in the RPE plays a role in CQ-induced retinopathy, chronic treatment of rats with CQ induces accumulation of POS-derived material both intracellularly and basal to the RPE.^8^^,^^9^

Studies of the effects of short-term treatment with relatively high doses of CQ (20–100 µm or more) on the RPE and other cells have reported not only elevation of lysosomal pH but also multiple effects on biosynthetic, endolysosomal, phagosomal, and autophagic pathways.^10^^–^^14^ Chronic CQ treatment, in contrast, in vivo and in vitro can result in lysosome adaptation and recovery of acidic pH.^15^^,^^16^ Lysosomal degradative capacity is maintained by the lysosome cycle, whereby, after fusion with cargo-carrying organelles, the lysosome must be reformed in order to retain the capacity to participate in further rounds of fusion.^17^^–^^19^ When lysosome reformation is insufficient to maintain lysosomal degradative capacity, a signaling cascade initiated from the lysosome via the MiT-TFE family of transcription factors can upregulate lysosomal gene expression and the biogenesis of new lysosomes.^20^ High doses of CQ have previously been shown to activate or upregulate TFEB and lysosomal gene expression,^15^^,^^16^ but the effects of CQ on lysosome reformation are unclear.

In order to investigate the response of RPE lysosomes to CQ, we have used RPE models treated with low doses of CQ that allow at least partial recovery of lysosomal pH after initial neutralization. After chronic CQ treatment, endocytic, phagocytic, and autophagic cargo accumulated, and the processing of cathepsin D from the immature inactive proform to the active mature form was impaired. Enlarged endo-, auto-, and phago-lysosomes accumulated in CQ-treated cells, accompanied by progressive loss of accessibility of these structures to the endocytic pathway. Accumulation of pro–cathepsin D preceded any detectable CQ-induced changes in cathepsin D expression, suggesting that it is primarily due to reduced processing/degradation rather than TFEB-mediated changes in gene expression. Taken together, our results suggest that CQ-induced reduction in lysosome reformation and resultant reduction in lysosomal degradative capacity in the RPE could be a contributory factor in CQ-induced retinopathy.

## Methods

### Cell Culture

The human embryonic stem cell (hESC) line (H9; WiCell, Wisconsin, USA) was maintained in mTeSR1 medium on Matrigel-coated plates (BD Biosciences, New Jersey, USA) before guided differentiation over 14 days as previously described.^21^^,^^22^ At day 14, immature RPE were obtained and seeded at 1 × 10^5^ cells/cm^2^ on growth factor–reduced Matrigel, as P0. After maturation in X-VIVO 10 medium (Lonza, Basel, Switzerland) for 30 days, pigmentation was evident and cells were passaged again (P1) to further expand the culture. From this point on, cells were plated onto laminin-coated surfaces (rhLaminin-521; BioLamina, Sundbyberg, Sweden). Cells were used after a minimum of 14 days in culture when they had achieved cobblestone morphology, differentiated apical and basolateral borders, and expressed multiple RPE cell markers (Supplementary Fig. S1) and, when cultured on trans-well filters, had transepithelial electrical resistances (TEERs) of >200 ohms/cm^2^.

Porcine RPE were isolated and cultured as previously described.^15^ All cells were used at P1 or P2 and, after culture in Dulbecco's modified Eagle's medium (DMEM)/10% fetal bovine serum (FBS) to reach confluence, were transferred to DMEM/1% FBS for 2 to 4 weeks. Cells displayed typical cobblestone morphology and differentiated apical and basolateral borders (Supplementary Fig. S1) and, when cultured on trans-well filters, achieved TEERs of >500 ohms/cm^2^.

ARPE-19 cells up to passage 30 were maintained in DMEM/10% FBS and for experiments were cultured in DMEM/10% FBS for 1 week to reach confluence before reduction to DMEM/1% FBS for 10 to 14 days. Cells displayed patchy areas of cobblestone morphology (Supplementary Fig. S1).

### POS Phagocytosis

POSs were isolated and purified from porcine eyes as previously described.^23^ The POS suspension (200 µg/mL) was sonicated for 10 minutes before adding to cells for 4 hours at 37°C in DMEM/10% FBS.

### Fluorescence Experiments

For drug and fluorescent reporters, see Table 1. After incubation for the indicated times, cells were washed three times with PBS and then analyzed by either flow cytometry or live-cell microscopy. For flow cytometry, cells were trypsinized and resuspended in FACS buffer (1% FBS and 2 mM EDTA in PBS). Acquisition was performed in a FACS CANTO II (BD Biosciences) flow cytometer, and at least 20,000 cells were analyzed using FlowJo version 10.1r7 software (Oregon, USA). For microscopy, cells were analyzed using a Leica SP8 (Milton Keynes, UK) or a Zeiss 710 (Cambridge, UK) confocal microscope, and digital images were analyzed using LSM Image software (Leica, Milton Keynes, UK) or ImageJ (National Institutes of Health, Bethesda, MD, USA). For immunofluorescence, cells were fixed with 4% paraformaldehyde (PFA), permeabilized, and blocked with 0.05% to 0.2% saponin or 0.02% Triton X100 in PBS/1% BSA for 30 to 60 minutes at room temperature. Cells were incubated with primary antibodies (Table 2) for 1 hour at 37°C or overnight at 4°C and detected with secondary antibodies conjugated with Alexa fluorophores (Invitrogen, Carlsbad, CA, USA). Cells were mounted in ProLong Gold Antifade Mountant with DAPI (ThermoFisher, Massachusetts, USA) and analyzed as above.

**Table 1.** Drugs and Fluorescent Reporters Used in This Study

Compound	Supplier	Vehicle	Final Concentration Used
Chloroquine	Sigma	PBS	As indicated in the text
Bafilomycin A	Sigma	DMSO	50 nm
Cycloheximide	Sigma	DMSO	2.5 µg/mL
Leupeptin	Sigma	PBS	1 µg/mL
Pepstatin	Sigma	DMSO	20 µg/mL
Lysotracker	ThermoFisher		50 nm
DQ-BSA	ThermoFisher		10 µg/mL
Dextran Alexa Fluor 555/488 (10,000 daltons)	ThermoFisher		0.5 µg/mL

**Table 2.** Primary Antibodies Used in This Study

Antigen	Antibody	Supplier	Cells	Application	Dilution
Cathepsin D	AF1014	R&D Systems	pRPE, ARPE19	WB	1:100
				IF	1:100
			hESC-RPE	IEM	1:100
			hESC-RPE	WB	1:1000
HSc70	Sc-7298	Santa Cruz Biotechnology	pRPE	WB	1:10,000
LAMP1	Ab24170	Abcam	pRPE	WB	1:150
CD107a					
LAMP1	H4A3	DSHB	ARPE19	IF	1:100
LAMP1 (C54H11)	#3243	Cell Signaling	hEScRPE	WB	1:1000
TGN46	PA5-23068	Invitrogen	ARPE19	IF	1:100
RetP1	MA5-11741	Invitrogen	hEScRPE	IF	1:100
P62	sc-28359	Santa Cruz	hEScRPE	WB	1:1000
LC3	L8918	Sigma	hEScRPE	WB	1:500
Actin	AB0145	Sicgen	hEScRPE	WB	1:5000
Calnexin (CANX)	AB0041	Sicgen	hEScRPE	WB	1:5000

IEM, immunoelectron microscopy; IF, immunofluorescence; WB, western blot.

### Western Blot

After lysis, SDS-PAGE, and transfer to polyvinylidene difluoride (PVDF) or nitrocellulose membranes, membranes were blocked with 7% milk in Tris-buffered saline (TBS) containing 0.1% Tween 20 or TBS blocking buffer (Li-COR, Cambridge, UK). Primary antibodies (Table 2) were diluted in blocking buffer (or Li-COR TBS blocking buffer + Tween 0.2%), incubated overnight at 4°C, and detected with either horseradish peroxidase (HRP)–conjugated secondary antibodies (Dako, California, USA) or infrared secondary antibodies (Li-COR) and incubated for 1 hour at room temperature. HRP-labeled secondary antibodies were detected by enhanced chemiluminescence (ECL; Pierce, ThermoFisher, Massachusetts, USA) and either exposure of membranes to x-ray film (GE Healthcare, Illinois, USA) and development in an SRX 101A processor (Konica Minolta, Basildon, UK) or detection with a ChemiDoc system (BioRad, California, USA). Infrared secondary antibodies were detected using a Licor imager (Li-COR). Densitometric quantification of bands was performed in ImageJ.

### Quantitative Real-Time PCR

Total RNA was purified from cell monolayers using the RNeasy Mini Kit (Qiagen, Hilden, Germany), as per the manufacturer's instructions. Then, 1 µg total RNA was reverse transcribed to synthesize the cDNA template using the Superscript VILOTM cDNA synthesis kit (Invitrogen). Predesigned TaqMan probes (ThermoFisher) were acquired targeting LAMP1 (Ss03378990_u1), cathepsin D (Ss03379762_u1), and GAPDH (Ss03375629_u1), and quantitative RT-PCR (qRT-PCR) was performed as per manufacturer instruction using the TaqMan Fast Advanced Master Mix on a QuantStudio 6 Flex system (both ThermoFisher). Relative mRNA expression was calculated as ΔΔCt (using GAPDH as the housekeeping gene) and the ΔΔCt values represented as fold change from experimental control (NT).

### Sequential Pulses With Fluorescent Dextrans

ARPE19 cells on glass-bottom dishes (MatTek, Massachusetts, USA) were incubated with dextran–Alexa Fluor 555 in DMEM/10% FBS for 2 hours at 37°C. Media were replaced with DMEM/10% FBS for 2 hours at 37°C before incubating ±10 µg/mL chloroquine in DMEM/1% FBS for 20 hours (1 day) or 68 hours later (3 days) at 37°C (replacing media every 24 hours). Cells were then incubated with dextran–Alexa Fluor 488 in DMEM/10% FBS for 2 hours at 37°C. The media were replaced with DMEM/10% FBS for 1.5 hours at 37°C before adding Lysotracker Deep Red for 30 minutes at 37°C. Subsequent live-cell imaging was conducted at room temperature using a Leica SP8 confocal microscope.

### Experiments Using BSA-Gold

To determine the fate of BSA-gold–containing lysosomes after the addition of chloroquine, ARPE19 cells were incubated with BSA-gold (5 nm) at the recommended concentration (University Medical Center Utrecht) in DMEM/10% FBS at 37°C before chasing for 2 hours in DMEM/10% FBS at 37°C. The media were then replaced with 10 µg/mL CQ in DMEM/1% FBS for 0, 24, or 72 hours at 37°C (replacing media every 24 hours) and cells processed for transmission electron microscopy (TEM).

To determine the impact of chloroquine treatment on delivery of BSA-gold to lysosomes, ARPE19 cells were incubated with BSA-gold for 2 hours at 37°C followed by 2-hour chase as above. The medium was then replaced with ±10 µg/mL chloroquine in DMEM/1% FBS for 20 hours (1 day) or 68 hours (3 days) at 37°C (replacing media every 24 hours). BSA-gold was then added in DMEM/10% FBS for 2 hours at 37°C before chase for 2 hours in DMEM/10% FBS at 37°C and cells processed for TEM.

### TEM

For conventional TEM, cells on Transwells were embedded as previously described^23^ and imaged on a JEOL 1010 (Tokyo, Japan) or a JEOL 1400 Plus TEM with an Orius SC1000B charge-coupled device camera with Digital Micrograph software (Gatan, California, USA).

Cryo-immuno TEM (Tokuyasu technique) was performed as previously described.^23^^,^^24^

### Statistics and Reproducibility

For reproducibility, an *N* ≥ 3 was attempted, but in a small number of cases due to experimental error, we resorted to an *N* = 2, where indicated. Experiments that were repeated on different days had results normalized to an internal control. Therefore, no standard error of the mean is given for the controls as these have been set to 1 for each repeat experiment. Statistical significance was determined by either one-way ANOVA, two-way ANOVA, or unpaired *t*-test. Statistical significance was not determined for any experiment with *N* < 3. The *N* and statistical tests used in each experiment are described in the figure legends.

## Results

In this study, we have used three different RPE cell models taking advantage of features of each that make them most suitable for different types of investigation. hESC-RPE began as our model of choice because they are genetically identical human cells that form polarized differentiated monolayers. However, they retain some embryonic features, including the biogenesis of melanosomes. Melanosomes are lysosome-related organelles, and immature melanosomes share constituents with lysosomes, confusing detailed analysis of effects of CQ on lysosomes. While melanization is frequently viewed as a marker of successful RPE differentiation, it is worth noting that there is little melanogenesis in adult RPE. Primary porcine RPE (pRPE) represent the best model of adult RPE cells but can only be passaged once to avoid dedifferentiation, meaning that they need to be repeatedly and freshly isolated from genetically variable sources. We have therefore also used the RPE cell line ARPE19 under conditions where it is less well differentiated than hESC-RPE and pRPE, but the lysosome distribution and content resemble that of pRPE. Clearly, it is important to demonstrate comparable responses to CQ in the three model systems, so we characterized short-term lysosome responses to CQ in all three models. We then used hESC-RPE given a daily regimen of POS feeding to analyze longer-term effects of CQ on lysosomal activity and protein levels. To probe the reasons underlying CQ effects required electron microscopic analysis of lysosomal compartments, as well as quantitative fluorescence analysis of lysosomal cargo delivery, and so we used pRPE and ARPE19 cells. Importantly, key responses to CQ, including expansion of acidic compartments and accumulation of lysosomal proteins before changes in mRNA levels, were reproduced across all three models.

### Low-Dose CQ Caused Initial Lysosome Neutralization Followed by Recovery and Lysosomal Expansion

**Figure 1.**
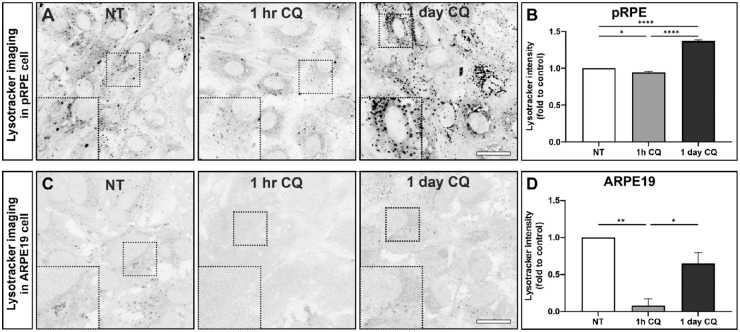
CQ-induced transient lysosome neutralization followed by lysosome adaptation. ARPE19 cells were incubated with 10 µg/mL CQ for the indicated times, and lysotracker was added for the final 30 minutes of incubation. (**A**, **C**) Confocal slices through live cells at the level of the nucleus are shown with contrast inverted (**A**, pRPE; **C**, ARPE19). *Insets* show *boxed areas* at higher magnification. *Scale bar*: 20 µm. (**B**, **D**) Quantitation of lysotracker intensity integrated over entire images. Results are mean ± SEM of three independent experiments. Statistical significance determine by one-way ANOVA; *P < 0.05, **P < 0.01.

Lysotracker is a fluorophore-linked weak base that freely crosses membranes at neutral pH but becomes protonated in acidic compartments and so specifically stains acidic organelles. After 1 hour of CQ treatment, lysotracker fluorescence was reduced to varying extents in pRPE, ARPE19 cells, and hESC-RPE, indicating reduction in lysosomal acidity, but after 24 hours, lysotracker intensity had at least partially recovered (ARPE19) or increased (pRPE and hESC-RPE) (Fig. 1 and Supplementary Fig. S2).

**Figure 2.**
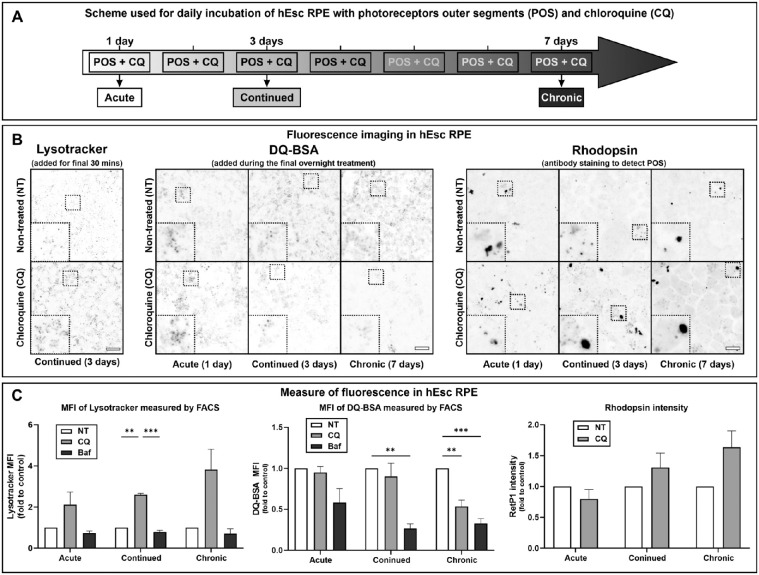
Modeling RPE responses to CQ: partial dysfunction of expanded lysosome compartment. (**A**) Scheme used for acute, continued, and chronic CQ treatments of hESC-RPE. Each POS incubation was 4 hours in 10% FBS followed by overnight chase in 1% FBS ± 5 µg/mL CQ or 50 nm Baf-A (acute), repeated three times (continued) and seven times (chronic). (**B**) CQ-treated cells were incubated with either lysotracker (added for the final 30 minutes of the treatment protocol) or DQ-BSA (added during the final overnight chase) and imaged live or were fixed, permeabilized, and stained with antirhodopsin antibody. Images are confocal slices with contrast inverted. *Scale bar*: 20 µm. (**C**) Quantitation of lysotracker and DQ-BSA mean fluorescence intensity by flow cytometry and rhodopsin intensity by confocal microscopy. Results are mean ± SEM of ≥2 independent experiments (chronic *N* = 2, hence excluded from statistical analysis) for mean fluorescence intensity (MFI) of lysotracker, ≥4 independent experiments for MFI of DQ-BSA, and ≥3 independent experiments for rhodopsin intensity. Statistical significance determine by two-way ANOVA; **P < 0.01, ***P < 0.001.

The adaptation to CQ treatment after 24 hours prompted us to investigate longer-term effects of CQ. To investigate these effects in more detail, we initially selected hESC-RPE as a well-differentiated genetically homogeneous cell line. Given the daily degradative burden of phagocytosed POS in vivo, hESC-RPE were incubated daily with POS for 4 hours, followed by 20-hour chase ± CQ. Acute treatment (single POS pulse and overnight chase) was compared with repeated daily POS pulses and overnight chases for 3 (continued) and 7 (chronic) days (Fig. 2A). Fluorescence microscopy showed the clear presence of lysotracker-positive puntae upon continued CQ treatment (Fig. 2B), and flow cytometry demonstrated an increase in mean fluorescence intensity of lysotracker with increasing length (from 1–7 days) of CQ but not bafilomycin A treatment (Fig. 2C). Importantly, a mildly acidic luminal pH is sufficient for lysotracker to become trapped within organelles where its fluorescence intensity is largely independent of pH. The increased fluorescence seen upon CQ treatment thus indicates expansion of acidic compartments. Bafilomycin is a V-ATPase inhibitor and most likely caused total dissipation of the pH gradient across lysosomal membranes.

Taken together, these data suggest that after transient lysosome neutralization, CQ induces a progressive expansion of the acidic lysosomal compartment that retains at least some activity.

### Degradation of Endocytic, Phagocytic, and Autophagic Cargo and Processing of Cathepsin D Is Inhibited in CQ-Treated hESC-RPE Cells

**Figure 3.**
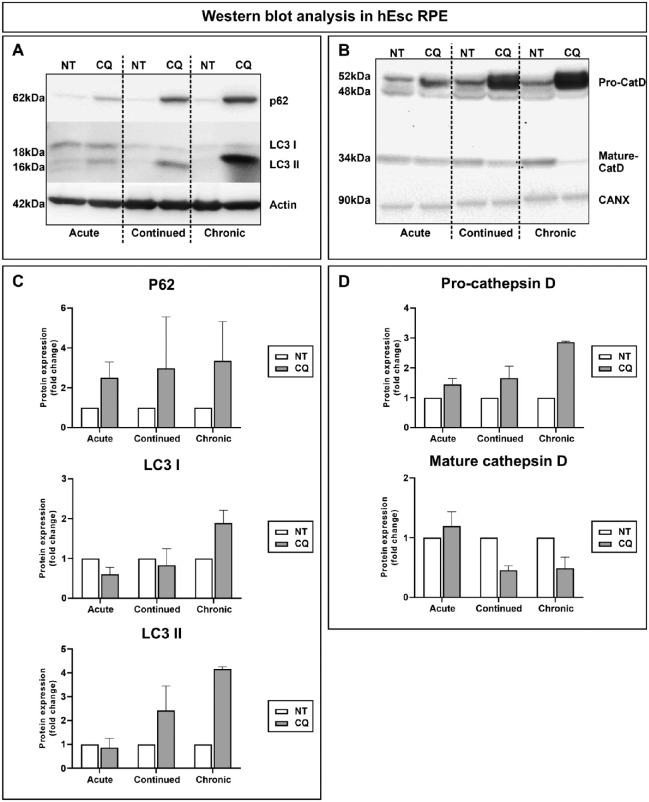
Modeling RPE responses to CQ: accumulation of autophagy markers and pro–cathepsin D. hESC-RPE were treated with POS and CQ as for Figure 2. Cell lysates were Western blotted for p62 and LC3 (**A**) and cathepsin D (**B**) with quantitation using ECL, shown in **C** and **D**, respectively. Results are mean ± SEM of ≥2 independent observations.

DQ-BSA, a strongly self-quenched fluorescent BSA derivative, becomes de-quenched when degraded by lysosomal proteases. Thus, a reduction in lysosomal protease activity results in reduced DQ-BSA fluorescence. When DQ-BSA was included in the final overnight chase, DQ-BSA fluorescent punctae were clearly present after 1 day of CQ treatment but became less evident after longer CQ treatments (Fig. 2B). Flow cytometry showed a progressive reduction in mean fluorescence intensity at 3 and 7 days after treatment, although not as great as that induced by bafilomycin (Fig. 2C). Rhodopsin immunostaining showed that CQ treatment also led to accumulation of undegraded POS that increased with length of treatment (Figs. 2B, 2C). CQ also induced accumulation of the autophagy substrate, p62, and the lipidated LC3-11 (Figs. 3A, 3C). Cathepsin D (CatD) is trafficked as a proform via the endocytic pathway to the lysosome, where it undergoes low pH-dependent processing to the mature form. Pro-CatD rapidly accumulated in CQ-treated cells, accompanied by slow depletion of the mature enzyme (Figs. 3B, 3D).

Inhibition of cargo degradation and CatD processing could be explained by CQ-induced partial loss of lysosome activity but could also be caused by reduced lysosomal delivery. To determine where undegraded cargo accumulated, CQ-treated hESC-RPE were examined by TEM, which showed accumulation of structures suggestive of autolysosomes (the product of autophagosome–lysosome fusion) and phagolysosomes (containing undegraded or partially degraded POS) (Supplementary Fig. S3). Immunoelectron microscopy (ImmunoEM) showed greatly increased CatD staining, most likely representing the proform, in enlarged vacuoles (Supplementary Fig. S4). “Classical” lysosomes are difficult to identify even in control hESC-RPE, partly because these cells retain some embryonic characteristics, including active biogenesis of melanosomes, which share constituents with lysosomes. We therefore turned to adult primary porcine RPE and the more experimentally tractable ARPE19 cell line, in order to determine the effects of CQ on lysosomal delivery in RPE cell models without the confounding effects of melanosome biogenesis.

### Adult Porcine RPE and ARPE19 Cells Also Exhibit CQ-Induced Accumulation of Pro–Cathepsin D and Lysosomal Expansion, Even in the Absence of Phagocytosed POS

**Figure 4.**
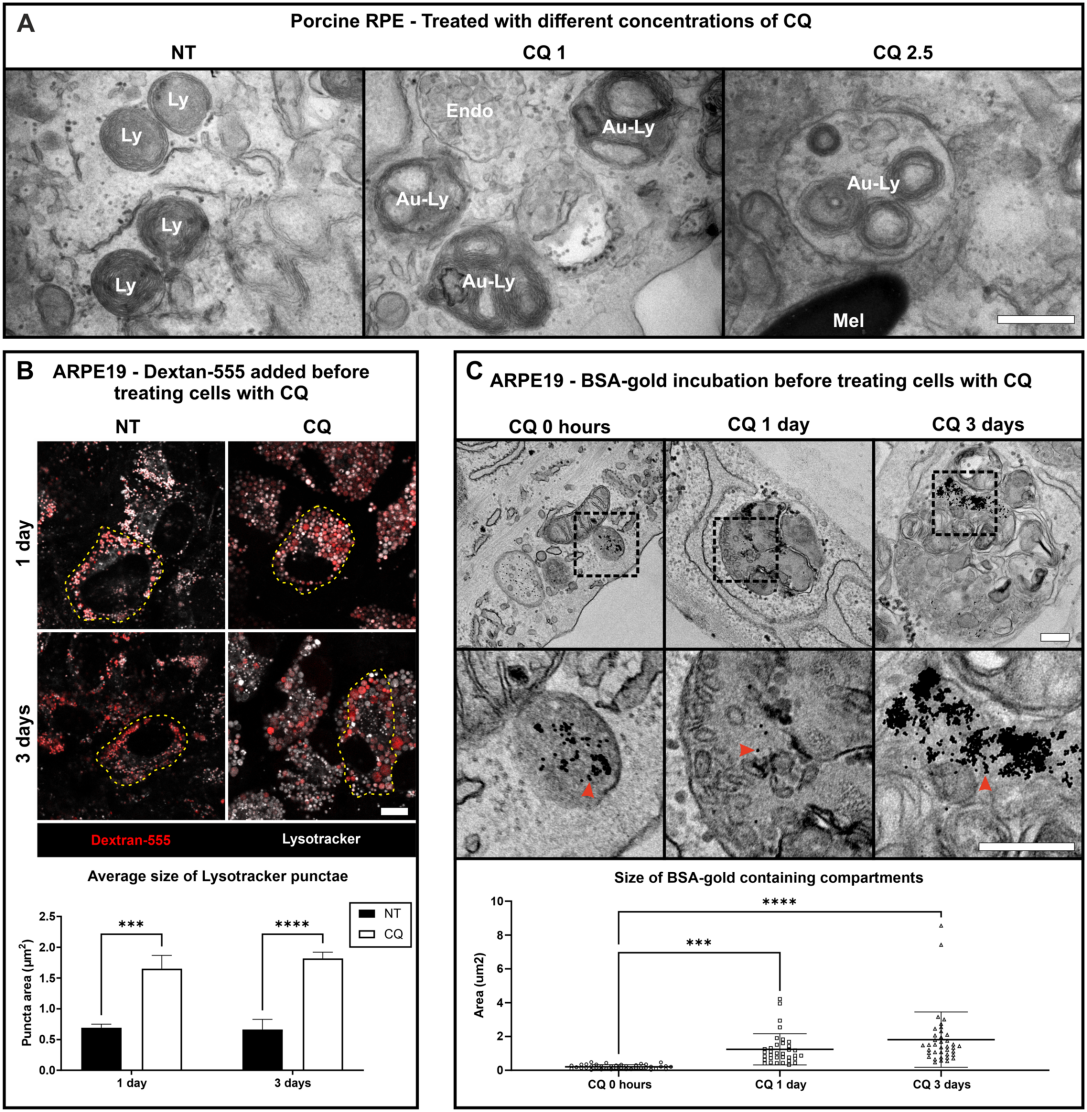
Expanded endolysosomes and autolysosomes in response to CQ in primary porcine RPE and ARPE19 cells. (**A**) Primary pRPE were incubated with the indicated concentrations (µg/mL) of CQ overnight and then processed for TEM. Examples of endosomes (Endo), lysosomes (Ly), autolysosomes (Au-Ly), and melanosomes (Mel) are indicated. *Scale bar*: 500 nm. (**B**) ARPE19 cells were incubated with a 2-hour pulse of dextran 555 in the absence of CQ before chase in the presence or absence of 10 µg/mL CQ for 1 or 3 days. Lysotracker was added for the final 30 minutes of incubation. *Scale bar*: 10 µm. The diameter of lysotracker-positive organelles was measured. Results are mean ± SEM of three independent experiments. Statistical significance was determined by two-way ANOVA; ***P < 0.001, ****P < 0.0001. (**C**) ARPE19 cells were incubated with a 2-hour pulse of BSA-gold (5 nm) in the absence of CQ and either fixed (0 hours) or chased with 10 µg/mL CQ for 1 or 3 days before fixing and processing for TEM. *Red arrowheads* indicate gold particles. The diameter of gold-containing organelles was measured. Results are mean ± SEM of ≥35 BSA-gold–containing organelles. Statistical significance was determined by one-way ANOVA; ***P < 0.001, ****P < 0.0001. *Scale bar*: 250 nm.

pRPE were particularly sensitive to CQ and so were given only overnight treatments of up to 2.5 µg/mL while ARPE19 cells readily tolerated 10 µg/mL CQ for at least 3 days. Western blotting revealed that CQ treatment induced pro-CatD accumulation in pRPE and ARPE19 cells, as in hESC-RPE, even in the absence of POS feeding (Supplementary Fig. S5). TEM revealed that pRPE contain readily identifiable electron-dense lysosomes, many of which also contain membrane whorls (Fig. 4A and Supplementary Fig. S6). After overnight CQ treatment, the lysosomes of pRPE were enlarged, and sometimes, particularly at higher concentrations, enlarged lysosomes with autophagic-like content, identified as autolysosomes, were observed (Fig. 4A and Supplementary Fig. S6), even in the absence of POS feeding. In order to unequivocally identify lysosomes, ARPE19 cells were incubated before CQ treatment with a 2-hour pulse of dextran 555 or BSA-Au followed by a 2-hour chase, a protocol we previously used to specifically load lysosomes.^25^^,^^26^ TEM analysis pre-CQ treatment revealed that the majority of BSA-gold was in electron-dense lysosomes, where it was aggregated due to degradation of the BSA that stabilizes the gold (Fig. 4C and Supplementary Fig. S7A). In cells treated with CQ (after preloading lysosomes with dextran or BSA-gold), dextran and lysotracker-positive lysosomes were significantly larger after 1- and 3-day treatment (Fig. 4B). TEM revealed progressive enlargement of BSA-gold–containing vacuoles after 1- and 3-day CQ treatment (Fig. 4C). After 1 day of treatment, electron-dense lysosomes containing aggregated BSA-gold were clearly enlarged while by 3 days, many BSA-gold–containing vacuoles had autophagic-like content, indicating that they were autolysosomes (Fig. 4C and Supplementary Fig. S7A).

**Figure 5.**
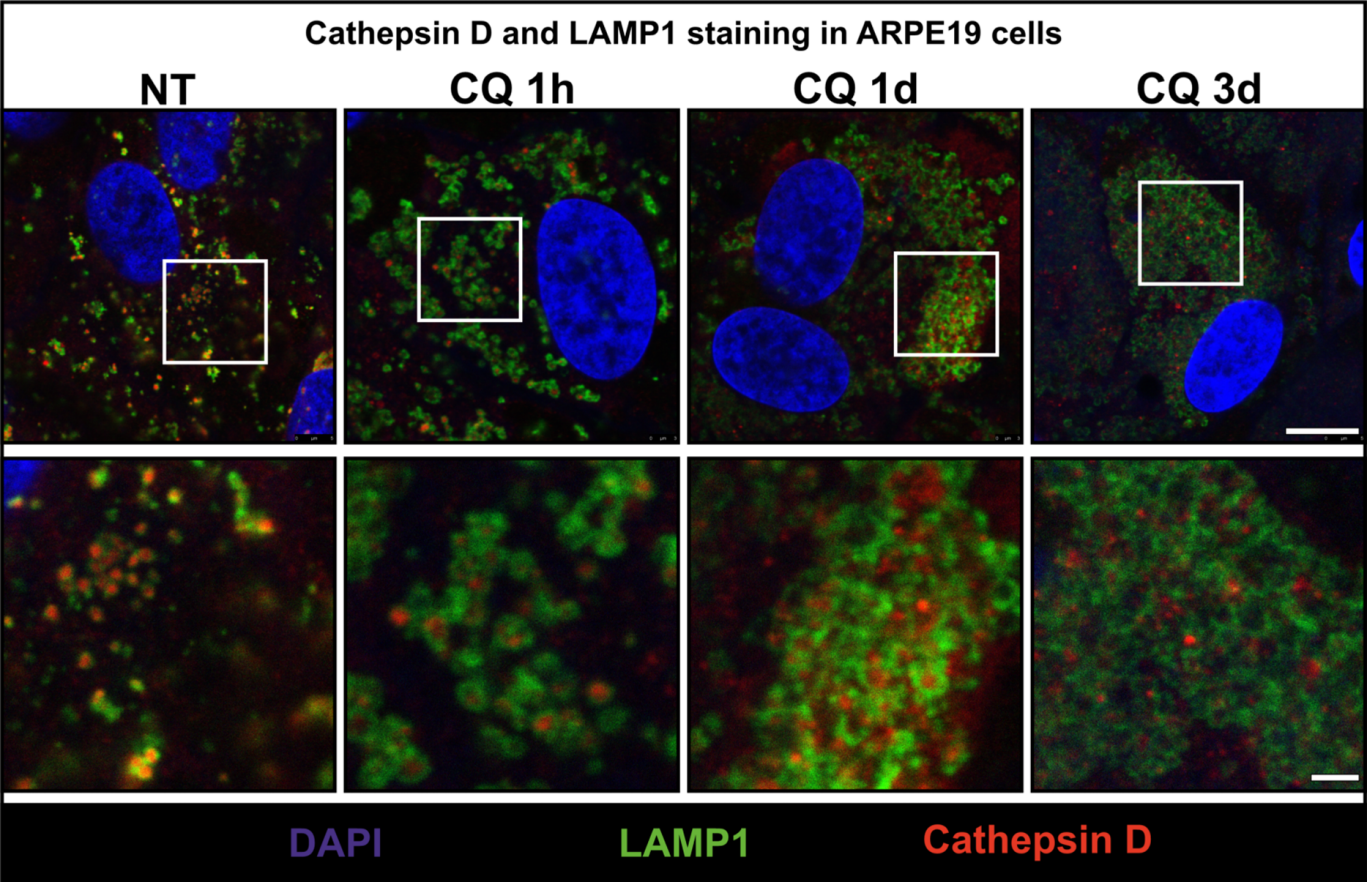
Distribution of cathepsin D and LAMP1 in CQ-treated ARPE19 cells. ARPE19 cells were incubated with 10 µg/mL CQ for up to 3 days before fixing and staining for LAMP1 (*green*) and cathepsin D (*red*). Confocal slices sectioning through the nucleus (*blue*) is shown. *Scale bars*: 10 µm (*top panels*) and 2 µm (*bottom panels*).

Consistent with the TEM studies, immunofluorescence of CQ-treated ARPE19 cells revealed a progressive enlargement of lysosomes positive for the lysosomal membrane protein, LAMP1 (Fig. 5). As LAMP1 is largely confined to the lysosomal limiting membrane and CatD is present within the lumen, there is only limited costaining between LAMP1 and CatD even in control cells. The separation of the two labels (lumen versus limiting membrane) is even more clearly resolved after 1 hour to 1 day of CQ treatment. However, although CatD staining could be clearly observed within LAMP1-positive structures in control cells and up to 1 day after CQ treatment, after 3 days, CatD-positive punctae were present that were not enclosed within LAMP1 labeling, suggesting that prolonged CQ treatment could induce accumulation of pro-CatD in prelysosomal compartments (Fig. 5). However, CQ could affect lysosomal delivery of LAMP1 as well as pro-CatD, raising the question of whether LAMP1 can be regarded as a bone fide lysosome marker in CQ-treated cells. Immunofluorescence with antibodies to the *trans*-Golgi network marker TGN46 and CatD showed no evidence of Golgi disruption or retention of cathepsin D in the TGN in CQ-treated ARPE19 cells (Supplementary Fig. S8).

Taken together, these data revealed that CQ-induced lysosomal expansion and pro-CatD accumulation are conserved across different models and species and not solely a response to accumulation of undigested POS. The gradual accumulation of pro-CatD in a post-Golgi prelysosomal compartment, as well as the accumulation of vacuoles resembling autolysosomes, suggests a possible defect in lysosome reformation.

### Lysosomes Gradually Lose Access to the Endocytic Pathway With CQ Treatment

**Figure 6.**
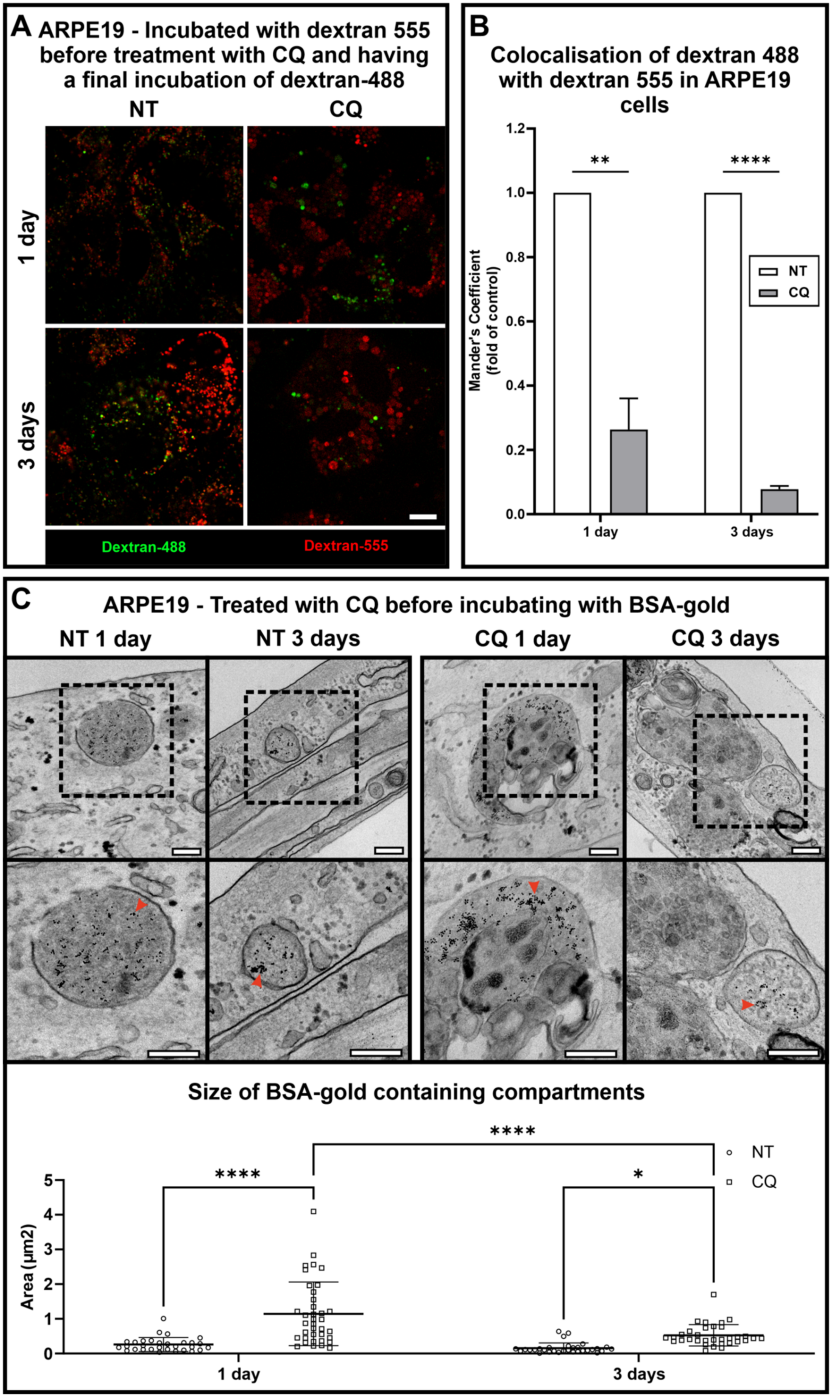
CQ induces progressive loss of accessibility of lysosomes to the endocytic pathway. (**A**, **B**) ARPE19 cells were incubated with a 2-hour pulse of dextran 555 in the absence of CQ before chase ± 10 µg/mL CQ for 1 or 3 days before a 2-hour pulse with dextran 488 followed by a 2-hour chase. Confocal slices are shown (**A**). *Scale bar*: 10 µm. (**B**) Quantitation of colocalization (Manders coefficient) of dextran 488 with dextran 555 expressed as fold to control. Results are mean ± SEM of three independent experiments. Statistical significance determined by unpaired *t*-test; **P < 0.01, ****P < 0.0001. (**C**) ARPE19 cells were incubated ±10 µg/mL CQ for 1 or 3 days before incubation with a 2-hour pulse of BSA-gold (5 nm) followed by a 2-hour chase before fixing and processing for TEM. *Red arrowheads* indicate gold particles. The diameter of BSA-gold–containing organelles was measured. Results are mean ± SEM of ≥30 BSA-gold–containing organelles. Statistical significance was determined by two-way ANOVA; ****P < 0.0001. *Scale bar*: 250 nm.

Lysosome reformation is necessary for lysosomes to maintain access to degradative cargo from the endocytic, phagocytic, and autophagic pathways. To determine whether CQ affects lysosome reformation, we determined whether endocytosed dextrans could reach lysosomes of CQ-treated ARPE19 cells. Lysosomes were preloaded with dextran conjugated to Alexa Fluor 555 (shown in red) and then treated ± CQ for 1 or 3 days before incubation with a second pulse of dextran conjugated to Alexa Fluor 488 (shown in green). In CQ-treated cells, there was a reduction in colocalization between the first and second dextran pulses, which was evident after 1 day and more marked after 3 days (Figs. 6A, 6B). This indicates that lysosomes present before CQ treatment became less accessible to newly endocytosed probes posttreatment. Importantly, uptake of the second dextran pulse was unaffected by CQ treatment (Supplementary Fig. S9). Some BSA-gold accessed lysosomes after CQ treatment, where it was partially aggregated, but particularly after 3 days, nonaggregated BSA-gold was also found in multivesicular bodies, the compartment that normally fuses with the lysosome (Fig. 6C and Supplementary Fig. S7). The size of organelles accessible to BSA-gold is significantly increased after 1 day of CQ treatment, indicating that at least some of the enlarged CQ-induced lysosomes remain accessible to endocytosed BSA-gold after 1 day of CQ treatment (Fig. 6C). After 3-day treatment, the size of BSA-gold–containing compartments, while greater than in untreated cells, is less that that after 1 day of CQ treatment. This suggests that the largest lysosomes that are induced by 3 days of CQ treatment are no longer accessible to newly endocytosed probes.

Taken together, these data suggest a defect in lysosome reformation, causing lysosomes to drop out of the lysosome cycle, losing accessibility to the endocytic pathway.

### CQ-Induced Accumulation of Pro–Cathepsin D Is Reproduced by Treatment With Protease Inhibitors and Precedes Changes in mRNA Levels

**Figure 7.**
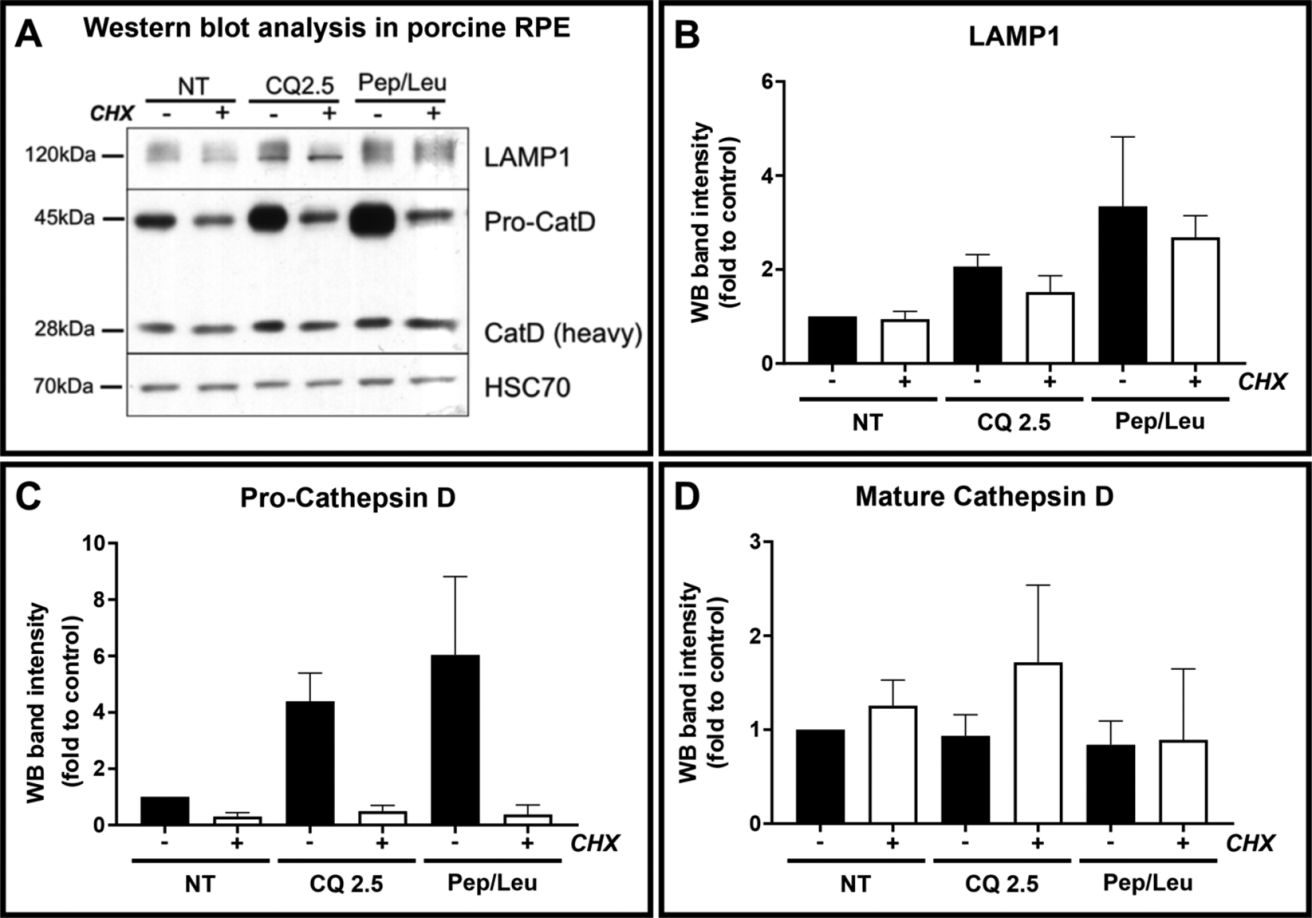
CQ-induced accumulation of pro–cathepsin D is reproduced by treatment with protease inhibitors. Primary pRPE were incubated overnight with or without CQ (2.5 µg/mL) or pepstatin/leupeptin, all in the presence or absence of cycloheximide (2.5 µg/mL). Cells were lysed and Western blotted for cathepsin D and LAMP1 (**A**) and quantitated using ECL (**B–D**). Results are mean ± SEM of at least three independent experiments. No statistical significance was determined by one-way ANOVA.

The marked CQ-induced accumulation of pro-CatD protein levels in all our models suggests that CQ upregulates CatD synthesis or a high natural rate of CatD synthesis leads to pro-CatD accumulation in CQ-treated cells because of reduced processing/degradation. To determine the effects of reduced processing/degradation, we compared the effects of the protease inhibitors pepstatin and leupeptin with that of CQ in the presence or absence of the protein synthesis inhibitor, cycloheximide. As global inhibition of protein synthesis is likely to have multiple effects, we used pRPE, which are sensitive to only overnight treatments with CQ. After overnight incubation, pRPE cells accumulated levels of pro-CatD and LAMP1 comparable to that induced by CQ, which was prevented by blocking protein synthesis (Fig. 7), consistent with a high natural rate of turnover of lysosomal constituents.

**Figure 8.**
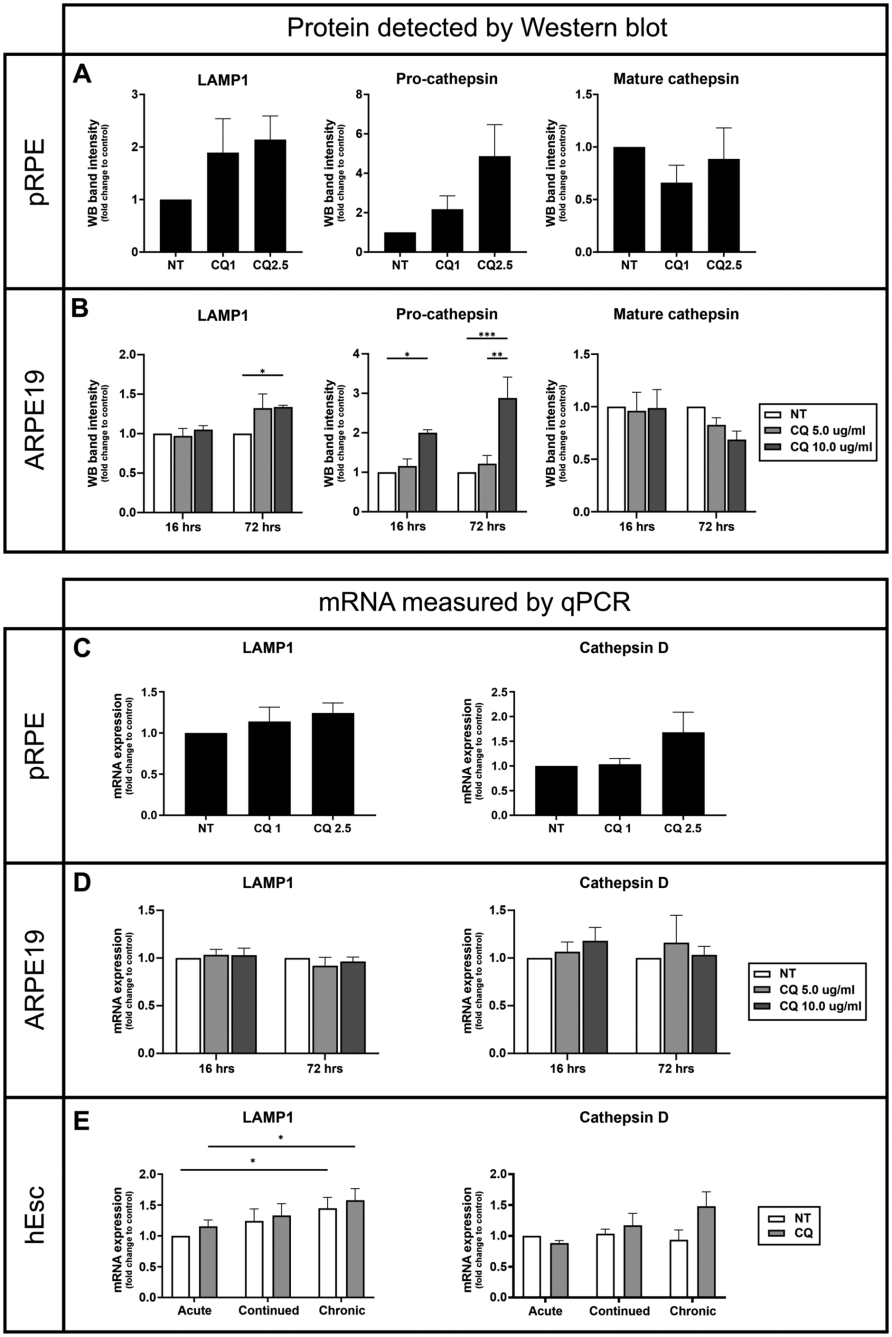
CQ-induced accumulation of lysosomal constituents precedes changes in lysosomal mRNA levels. (**A**, **B**) Primary pRPE and ARPE19 cells were incubated with the indicated CQ concentrations overnight (pRPE) or 16 to 72 hours (ARPE19 cells) and analyzed for LAMP1 and cathepsin D levels by Western blot. Representative gels are shown in Supplementary Figure S5. Results are mean ± SEM of three independent experiments using ECL and densitometric scanning of x-ray film for pRPE and infrared (Li-COR) detection for ARPE19 cells. (**C–E**) Primary pRPE and ARPE19 cells were incubated with the indicated CQ concentrations overnight (pRPE) or 16 to 72 hours (ARPE19 cells). hESC-RPE were incubated with CQ and POS as described in Figure 2. mRNA levels of LAMP1 and cathepsin D were measured by quantitative PCR and expressed as fold to untreated. Results are mean ± SEM of ≥3 observations. Statistical significance was determined by (**A**, **C**) one-way ANOVA and (**B**, **D**, **E**) two-way ANOVA; *P < 0.05, **P < 0.01, ***P < 0.001.

No changes in CatD or LAMP1 mRNA levels were detected in CQ-treated ARPE19 cells despite significant changes in protein levels at higher CQ doses (Fig. 8). Similarly, although there was a trend toward elevated lysosomal mRNA levels in pRPE and hESC-RPE at higher doses or prolonged treatments, respectively, these were not significant and undetectable at lower doses/shorter durations where CatD and LAMP1 protein accumulation was clear (Figs. 8 and 3).

Taken together, these data suggest that, at least after short- or low-dose CQ, the pro-CatD and LAMP1 accumulation is primarily due to their reduced processing/degradation. After prolonged treatment or higher doses, compensatory lysosome biogenesis may also occur.

## Discussion

The underlying cause of CQ-induced retinopathy is unclear, but the high dependency of the RPE on lysosomal degradation suggests that this cell type could be particularly sensitive to lysosomotropic agents like CQ. The cellular response to CQ depends on dose and duration. Confounding factors in assessing the response of RPE cells to CQ are the limitations of RPE cell models and the fact that melanin can sequester CQ, making the effective dose difficult to assess.^27^ Our aim was to use conditions of CQ treatment where lysosomes are the primary target. To generate an accelerated cellular model that most closely resembles human RPE cells in vivo we began by using hESC-RPE given a daily dose of POS and treated with low doses of CQ for increasing lengths of time. This allowed resolution of sequential events in the CQ response. After immediate lysosome neutralization, lysosomes at least partially reacidified and became enlarged. Progressive enlargement was accompanied by gradual accumulation of endocytic, phagocytic, and autophagic cargos, as well as pro-CatD, an enzyme that depends on lysosomal activity for its processing to the mature form. Together, this indicates that lysosome adaptation was only partial, and there was a progressive loss of lysosomal degradative capacity. However, unlike adult RPE cells in vivo, stem cell–derived RPE cells in culture continue to make melanosomes, resulting in highly melanized cultures containing large quantities of immature melanosomes. As melanosomes are lysosome-related organelles, this property made hESC-RPE a difficult model in which to analyze the lysosome cycle and new lysosome biogenesis. We therefore turned to primary pRPE, which retain characteristics of adult RPE in culture, and ARPE19 cells and found that CQ also induced lysosomal enlargement and accumulation of pro-CatD in these models, even in the absence of POS feeding. Although there were differences in dose responses of the different models, this indicates consistent responses to CQ across our different models and that the lysosomal enlargement was not solely caused by accumulation of undigested POS.

Reduced cargo degradation and CatD processing could reflect partial loss of lysosomal enzyme activity, most likely due to mildly elevated lysosomal pH. However, reduced cargo degradation and CatD processing could also be due to inhibition of lysosomal delivery. Indeed, CQ has been reported to inhibit autophagosome–lysosome fusion, although at considerably higher concentrations than in the present study.^12^ At least in the early stages of CQ treatment, in our study, cargo delivery to lysosomes was not blocked as BSA-gold could reach lysosomes in ARPE19 cells, where it was at least partially aggregated. Additionally, DQ-BSA degradation that depends on lysosomal delivery was unaffected after only overnight CQ treatment in hESC-RPE. However, sequential pulses of dextrans separated by increasing lengths of CQ treatment in ARPE19 cells demonstrated a gradual loss of accessibility of preexisting lysosomes to newly endocytosed probes. Newly synthesized CatD, like most lysosomal enzymes, is delivered to lysosomes via endosomes. Therefore, while initial accumulation of pro-CatD may be due to mildly elevated lysosomal pH, newly synthesized pro-CatD most likely progressively accumulates in endosomes in CQ-treated cells.

**Figure 9.**
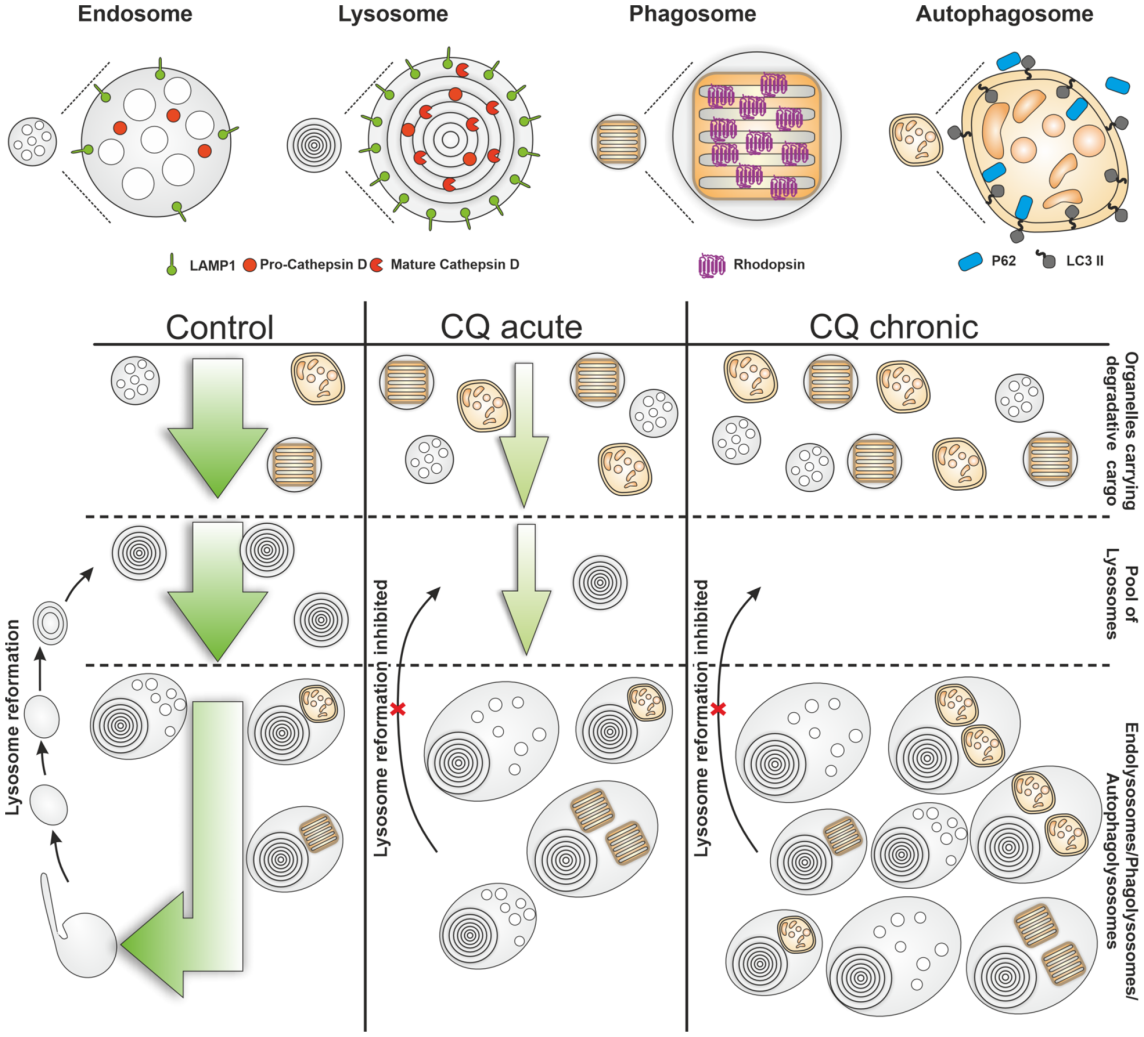
Schematic representation of lysosomal responses to chloroquine. In control cells, lysosomes readily reform after fusion with cargo-carrying vacuoles, replenishing the pool of LAMP1 and cathepsin D–positive lysosomes able to fuse with cargo-carrying vacuoles (endosomes, phagosomes, and autophagosomes). After CQ treatment, lysosome reformation is inhibited, resulting in accumulation of endolysosomes, phagolysosomes, and autolysosomes. In parallel, the pool of lysosomes able to fuse with cargo-carrying vacuoles becomes depleted, resulting in an accumulation of rhodopsin-positive phagosomes (in POS fed cells), autophagosomes, and endosomes that carry newly synthesized lysosomal constituents.

That lysosomes are initially able to receive cargo from the endocytic pathway after CQ treatment but gradually lose that accessibility is consistent with lysosome reformation being inhibited in CQ-treated cells, causing a gradual depletion of lysosomes capable of interacting with cargo-carrying organelles (Fig. 9). The process of lysosome reformation is incompletely understood. Tubulation from the limiting membrane of autolysosomes involves clathrin, PIKfyve, and KIF5B and precedes the budding of protolysosomes from tubule tips.^28^^,^^29^ Protolysosomes initially lack lysosomal enzymes and so must be repopulated either by interaction with existing lysosomes or delivery of newly synthesized lysosomal enzymes. Lysosome reformation from phagolysosomes and endolysosomes shares some molecular requirements with autolysosome reformation. After phagocytosis of apoptotic cells or bacteria, requirements for clathrin, PIKfyve, KIF5B, and tubulation have been reported.^30^^,^^31^ This latter reformation process also depends on the biosynthetic pathway so may also involve delivery of newly synthesized acid hydrolases.^30^ PIKfyve-dependent membrane remodeling has also been implicated in lysosome reformation from endolysosomes.^32^ The mechanism underlying inhibition of lysosome reformation in CQ-treated cells is unclear. Tubulation from the limiting membrane of autolysosomes is regulated by mammalian target of rapamycin complex (mTORC) signalling.^33^ Upon induction of autophagy, mTORC signaling is inhibited, but amino acids liberated through degradation of autophagocytosed content cause mTORC to be reactivated in a step necessary to allow autolysosome tubulation. CQ-induced mild elevation of lysosomal pH and resultant accumulation of incompletely processed autophagic cargos could modulate mTORC activation and inhibit lysosome tubulation. Importantly, most studies of lysosome reformation from autolysosomes have relied on induction of autophagy by amino acid starvation, while in the current study, we have shown that CQ has major effects on the lysosome cycle in nonstarved cells. A recent study indicates that delivery of PI(3)P to lysosomes provides the substrate for PIKfyve-dependent production of PI(3,5)P2 to support autolysosome reformation during basal autophagy.^34^ Analysis of the inositol phospholipid content of lysosomes in CQ-treated RPE would help to elucidate the mechanism underlying CQ-induced inhibition of lysosome reformation.

Treatment with CQ or targeting lysosomal proteases directly with protease inhibitors induced accumulation of newly synthesized pro-CatD and, to a lesser extent, the lysosomal membrane protein, LAMP1, without detectable changes in CatD or LAMP1 mRNA levels. Thus, at least initially, the accumulation of lysosomal proteins is due to reduced processing/degradation, rather than enhanced synthesis. The greater accumulation of pro-CatD, compared with LAMP, may reflect the hostile environment within the lysosomal lumen, requiring a high rate of basal turnover of lysosomal enzymes. That mature CatD was not rapidly depleted in CQ-treated cells suggests that CQ treatment inhibited the normal rate of degradation of this enzyme. The lysosomal membrane protein, LAMP1, forms part of the glycocalyx around the inner lysosomal membrane, protecting it from the hostile environment within. The accumulation of LAMP1 in CQ-treated cells may therefore reflect an inhibition of degradation of lysosomes themselves rather than lysosomal proteins. Damaged lysosomes are targeted for lysophagy via ubiquitination.^35^ CQ-induced inhibition of lysosome reformation might be expected to cause the accumulation of lysosomes normally cleared via lysophagy. Although considerable progress in identifying regulators of lysophagy has been made in recent years, most studies have relied on experimental induction of lysosomal damage,^35^ while the role of lysophagy in basal lysosome turnover is less well understood.

Lysosomotropic agents, including CQ, have been shown in the RPE and other cells to activate TFEB-mediated lysosomal gene transcription. Using doses of CQ lower than in most previous studies, we found that an earlier response to CQ was inhibition of lysosome reformation, thus taking preexisting lysosomes out of the lysosome cycle. A secondary response may be to upregulate CQ-induced lysosomal gene transcription in order to generate new lysosomes to replace those lost from the lysosome cycle. Considerable attention is being paid to upregulating TFEB-induced lysosome biogenesis in the brain, where accumulation of abnormal proteins and lipids plays a role in multiple neurodegenerative diseases.^36^ Upregulating TFEB-mediated activation of lysosomal gene transcription to reduce the potential danger of CQ-induced retinopathy would likely promote the biogenesis of lysosomes with the same impaired ability to reform as the preexisting lysosomes. An alternative approach might be to target lysosome reformation. The assay reported here using sequential pulses of dextrans to determine the accessibility of preexisting lysosomes to newly internalized probes could form the basis of a screen for drugs that promote lysosome reformation in CQ-treated RPE cells. This could open up an alternative potential way to enhance the lysosomal degradative capacity of the RPE in CQ-induced retinopathy as well as other retinal degenerative diseases characterized by accumulation of partially processed lysosomal cargoes.

## Supplementary Material

Supplement 1
