# Supplementary material for: Impaired Lysosome Reformation in Chloroquine-Treated Retinal Pigment Epithelial Cells
Source: Invest Ophthalmol Vis Sci. 2023 Aug 7;64(11):10. doi: 10.1167/iovs.64.11.10 (PMC10411645; doi:10.1167/iovs.64.11.10)
Supplement: Supplement 1 [file iovs-64-11-10_s001.pdf]

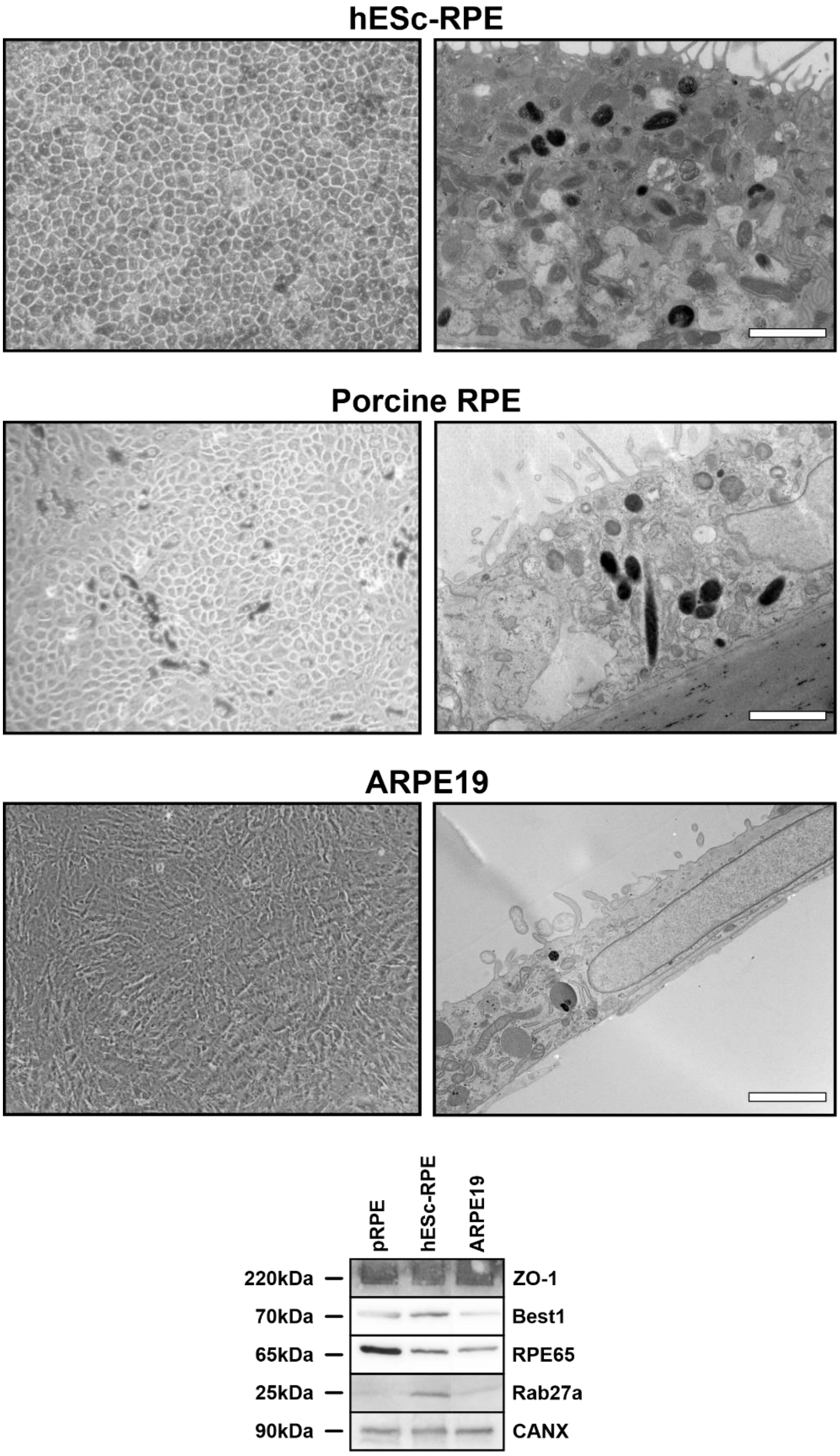

Supplementary Figure 1 Morphology and protein expression of hESC-RPE, primary porcine RPE and ARPE19 cells

hESC-RPE, primary porcine RPE and ARPE19 cells were analysed by bright field microscopy and transmission electron microscopy. hESC-RPE showed cobblestone morphology and differentiated apical surfaces. ARPE19 cells showed patchy cobblestone morphology but were less columnar than the other cell types. hESC-RPE were highly pigmented, primary porcine RPE contained less pigmentation and ARPE19 cells were not pigmented. All models express RPE markers Best1, RPE65 and Rab27a. Scale bar: 2µm

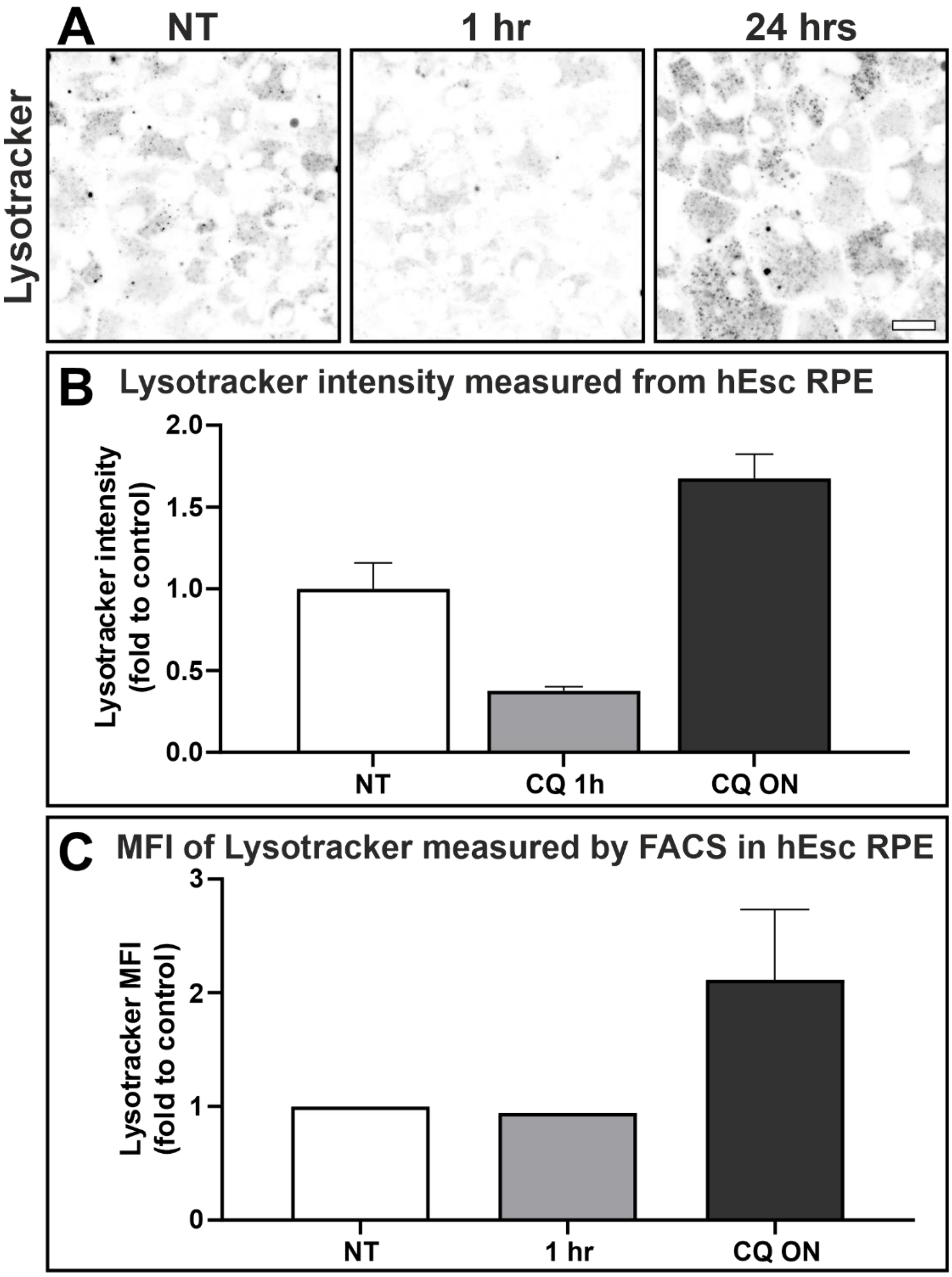

Supplementary Figure 2 CQ induced transient lysosome neutralization followed by lysosome adaptation in ARPE19 cells

hESC-RPE cells were incubated with 5μg/ml CQ for the indicated times and lysotracker was added for the final 30 minutes of incubation. A: Confocal slices through live cells. Scale bar: 20μm. B: Quantitation of lysotracker intensity. Results are means of 7 images per experimental condition. C: Quantitation of mean lysotracker intensity by flow cytometry. Results are means +/- SEM of 4 independent experiments for NT and CQ ON and a single experiment for 1hr.

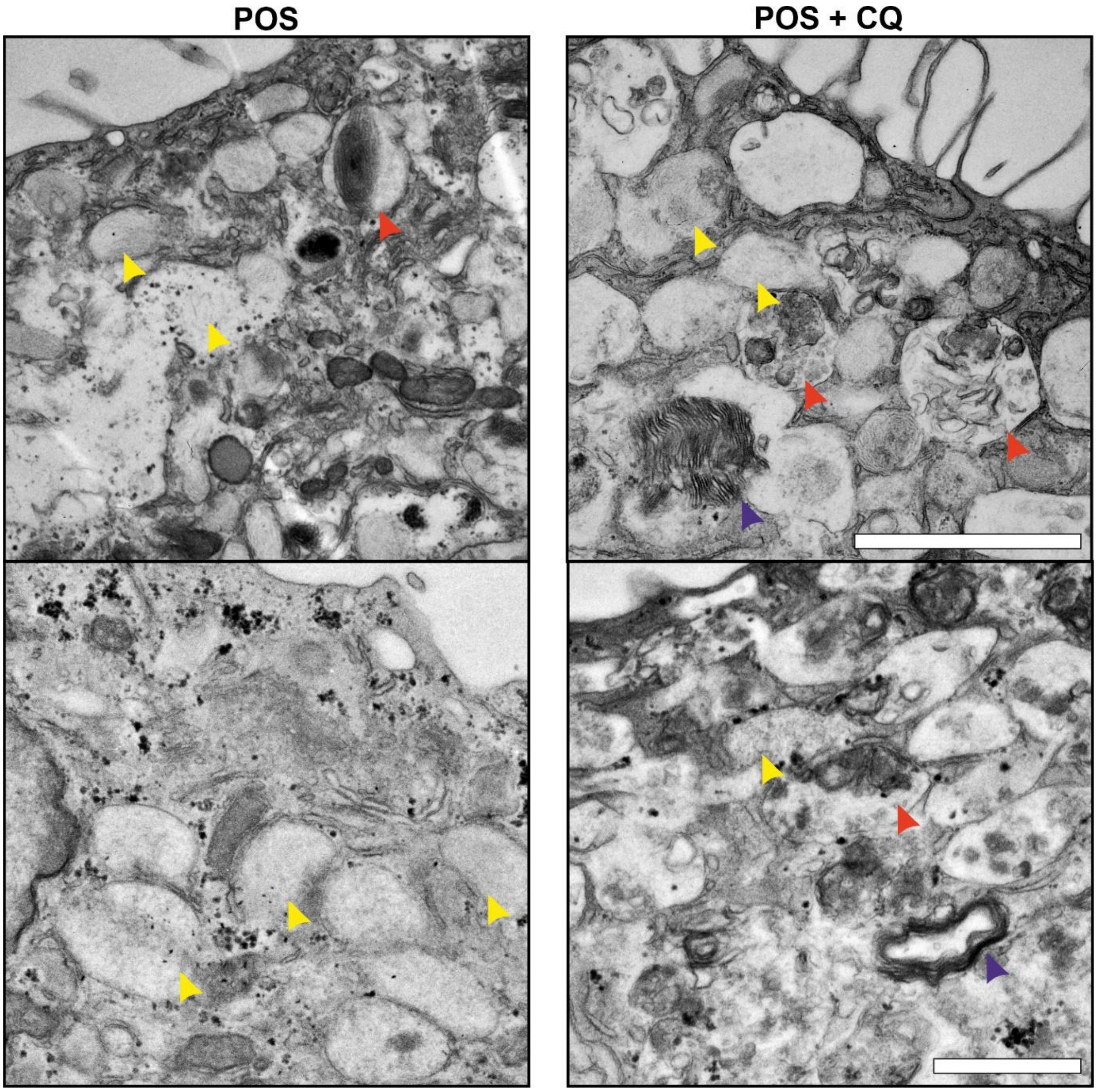

Supplementary Figure 3 EM characterisation of CQ-treated hESC-RPE

hESC-RPE were incubated with the continued (3 day) treatment with POS and CQ (as described for Figure 2) before fixing and processing for TEM. Red arrowheads indicate enlarged lysosome-like vacuoles whilst yellow arrowheads indicate vacuoles that could represent immature melanosomes. Purple arrowheads indicate structures that resemble phagocytosed POS. Scale bars: 2µm (top panels) 100nm (bottom panels).

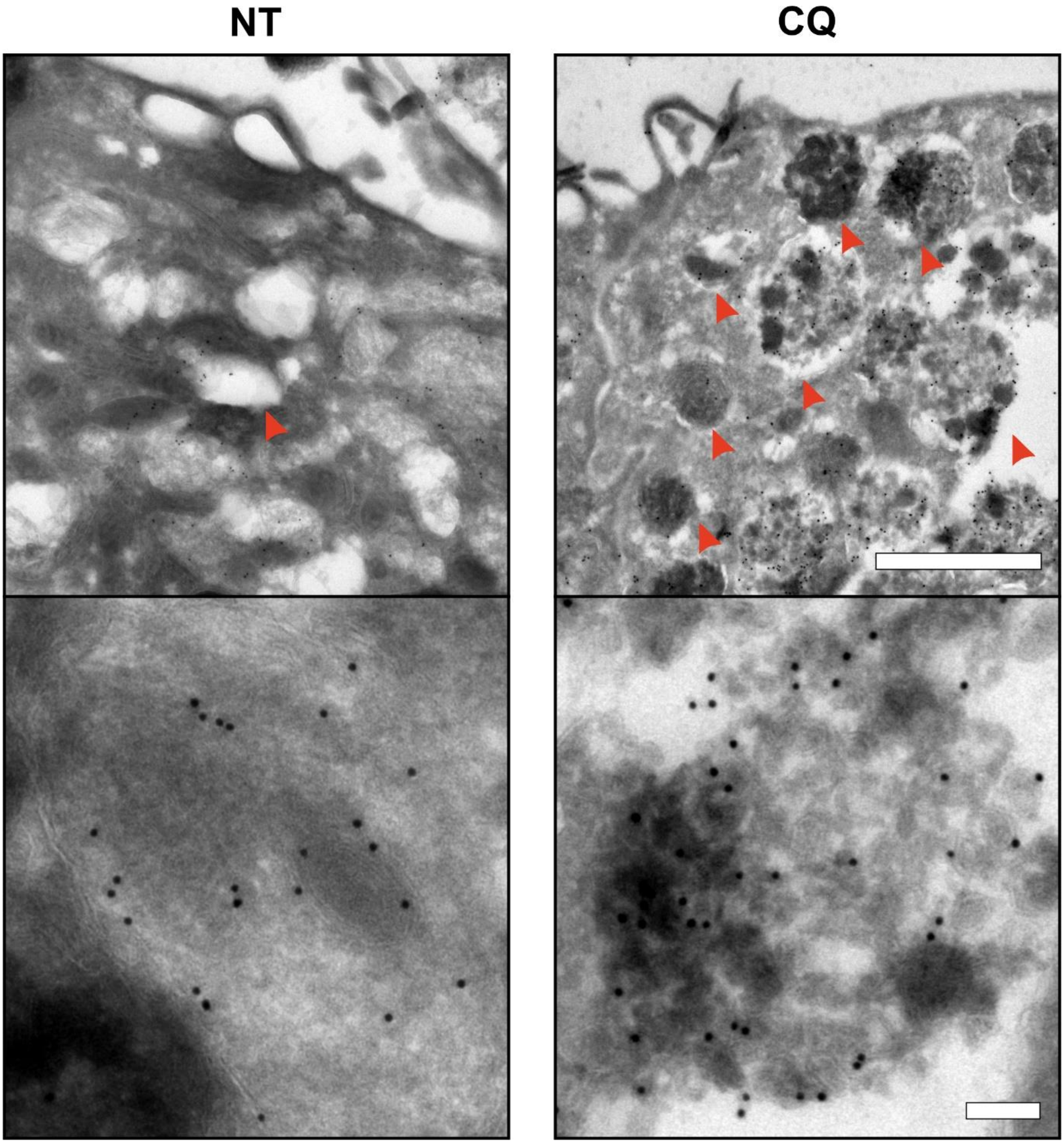

Supplementary Figure 4 Immuno-EM of cathepsin D in hESC-RPE

hESC-RPE were incubated with the continued (3 day) treatment with POS and CQ (as described for Figure 2) before fixing and processing for cryo-immunoEM. Thawed cryosections were labelled with anti-cathepsin D antibody and protein A gold. Red arrowheads indicate cathepsin D positive lysosome-like vacuoles. Scale bars: 1μm (top panels) 100nm (bottom panels)

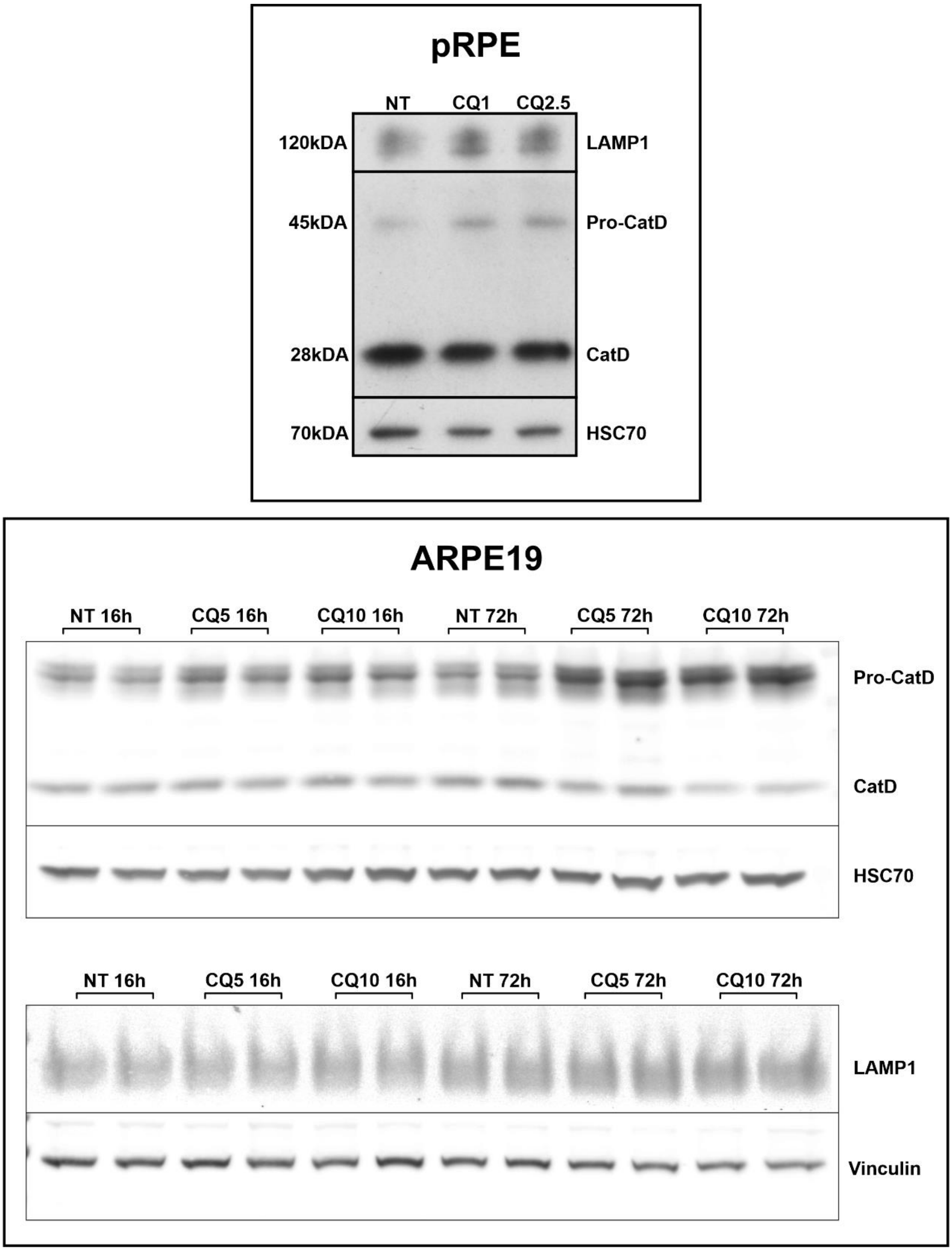

Supplementary Figure 5 Western blots of lysosomal proteins in CQ-treated pRPE and ARPE19 cells

Primary porcine RPE and ARPE19 cells were incubated with the indicated CQ concentrations (µg/ml) overnight (pRPE) or with 10µg/ml CQ for 16-72 hours (ARPE19 cells) and analysed for LAMP1 and Cathepsin D levels by Western blot, using ECL and densitometric scanning of X-ray film for pRPE and infra-Red (LiCor) detection for ARPE19 cells.

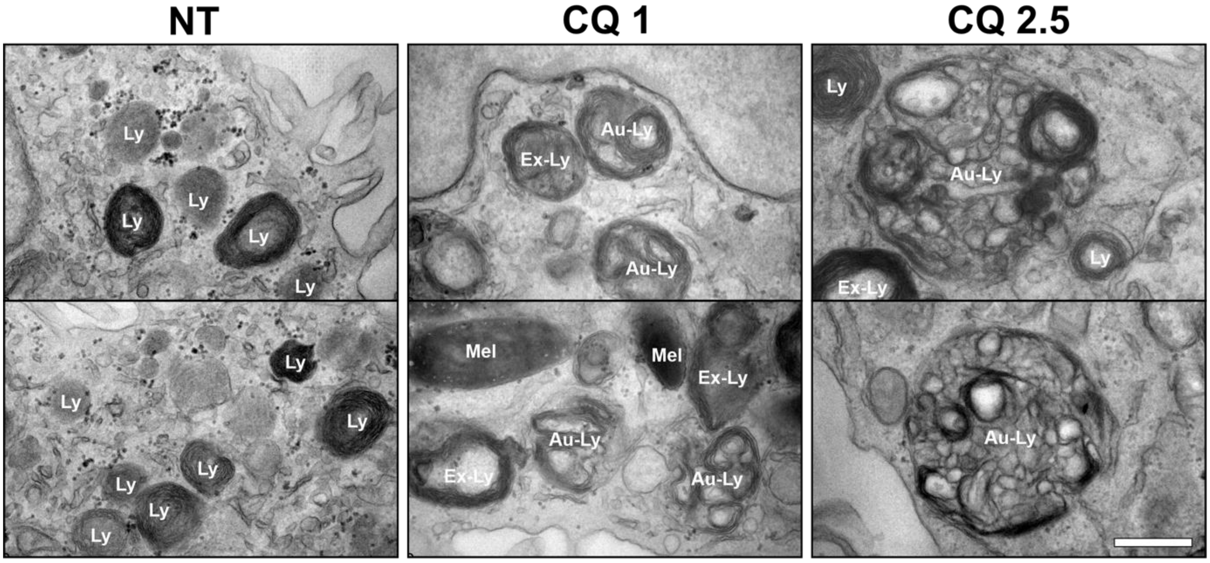

Supplementary Figure 6 Further examples of the effects of CQ on lysosome morphology in primary porcine RPE (see Figure 4)

A: Primary porcine RPE were incubated with the indicated concentrations (µg/ml) of CQ overnight and then processed for TEM. Examples of Lysosomes (Ly), melanosomes (Mel), expanded lysosomes (Ex-Ly) and autolysosomes (Au-Ly) are indicated. Scale bar: 500nm.

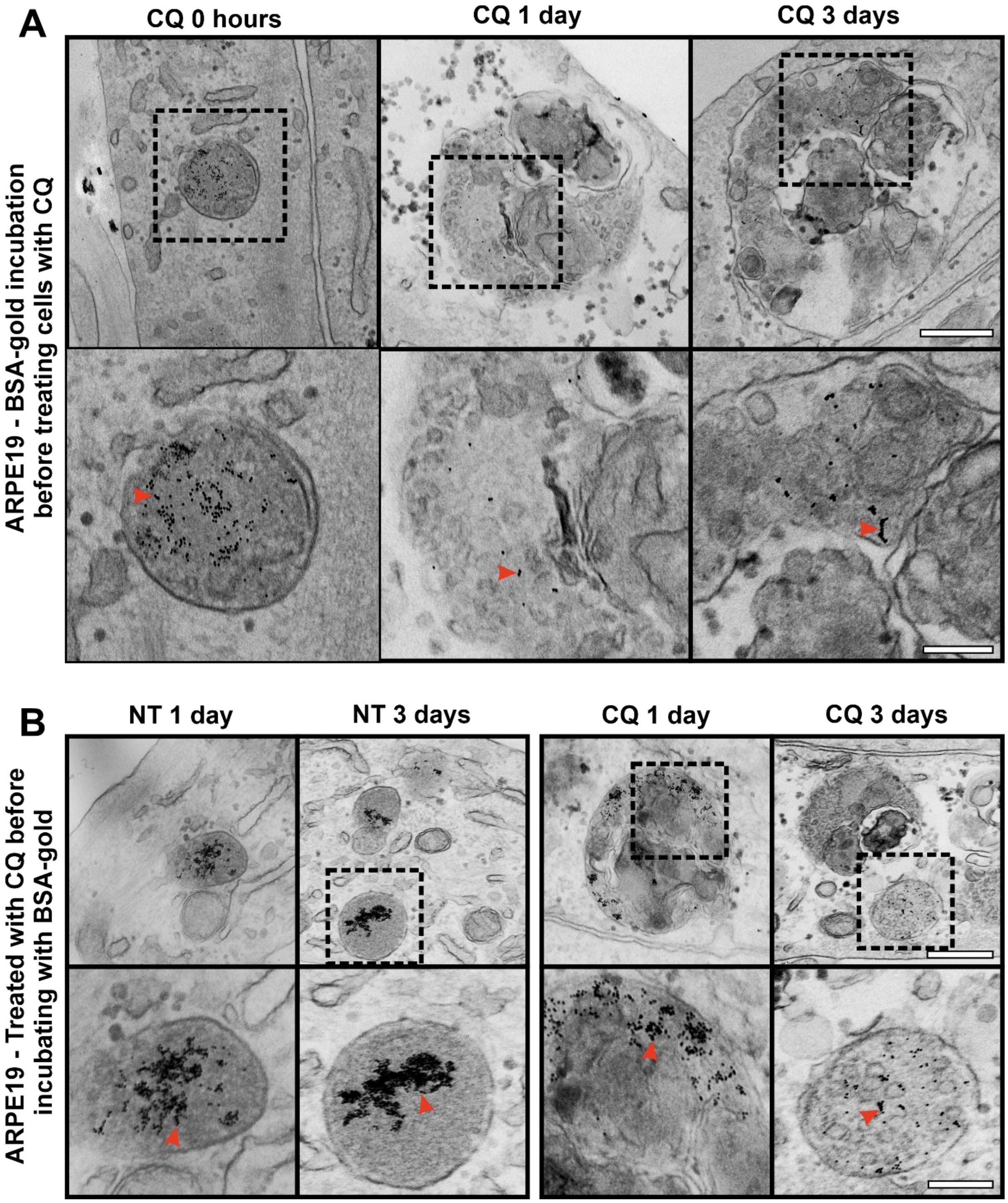

Supplementary Figure 7 Further examples of the effects of CQ on the delivery of BSA-gold to lysosomes and on the morphology of gold pre-loaded lysosomes (see Figure 6)

A: ARPE19 cells were incubated with a 2 hour pulse of BSA-gold (5nm) in the absence of CQ and were either fixed (0 hours) or chased with 10µg/ml CQ for 1 or 3 days before fixing and processing for TEM. Scale bars: 500nm (upper panels) and 200nm (lower panels). B: ARPE19 cells were incubated +/- 10µg/ml CQ for 1 or 3 days before incubation with a 2 hour pulse of BSA-gold (5nm) followed by 2 hour chase before fixing and processing for TEM. Boxed regions are magnified in the panel immediately below. Scale bars: 500nm (upper panels) and 200nm (lower panels). Red arrowheads indicate gold particles within the TEM images.

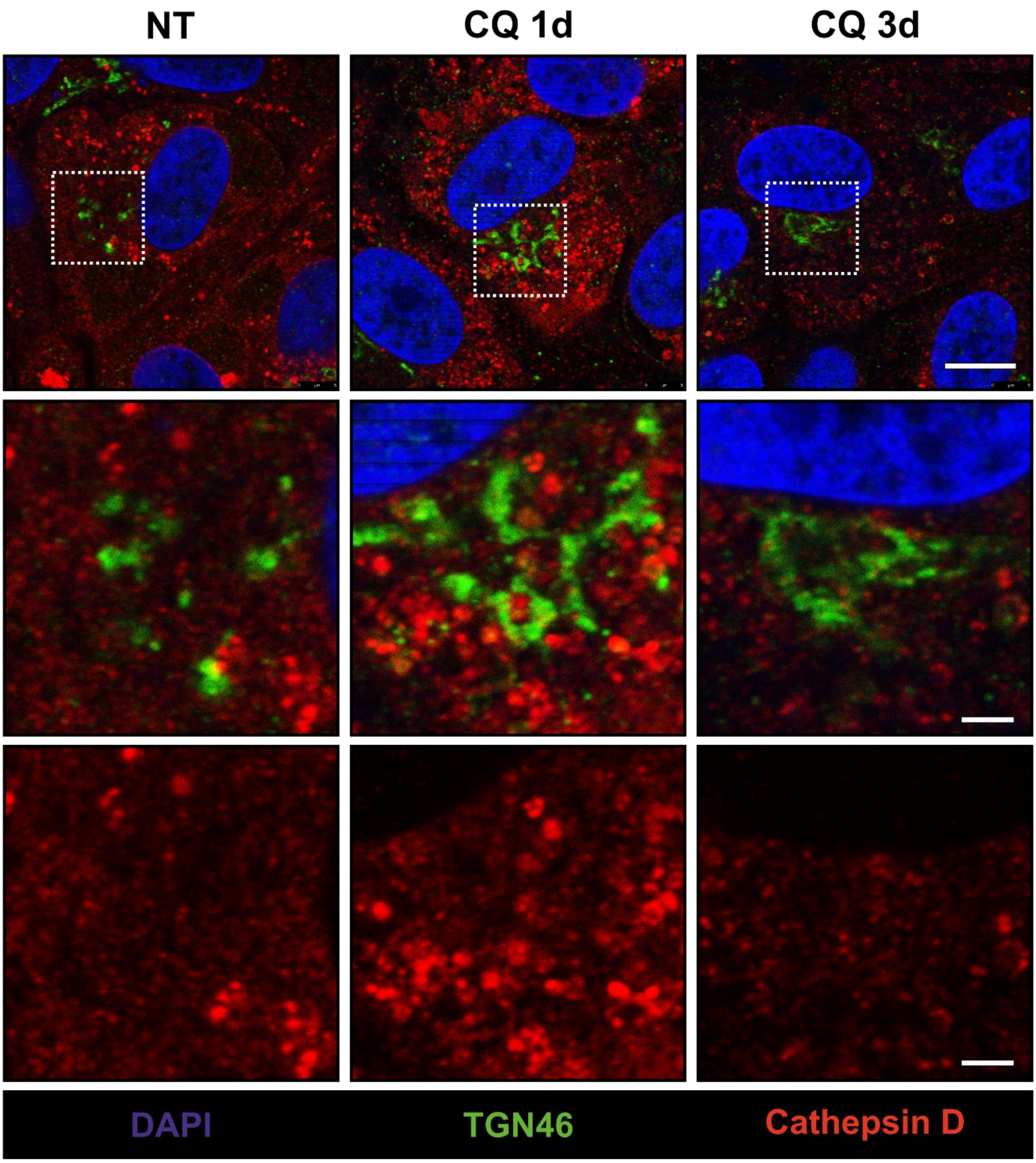

Supplementary Figure 8 Costaining of cathepsin D with TGN 46 in ARPE19 cells

ARPE19 cells were incubated with 10µg/ml CQ for up to 3 days before fixing and staining for LAMP1 (green) and cathepsin D (red). Confocal slices sectioning through the nucleus (blue) are shown. Single labelling of cathepsin D is shown in the bottom panels to show that there is little detectable cathepsin D staining in regions that stain for TGN46. Scale bars: 10µm (top panels), 2µm (middle and bottom panels).

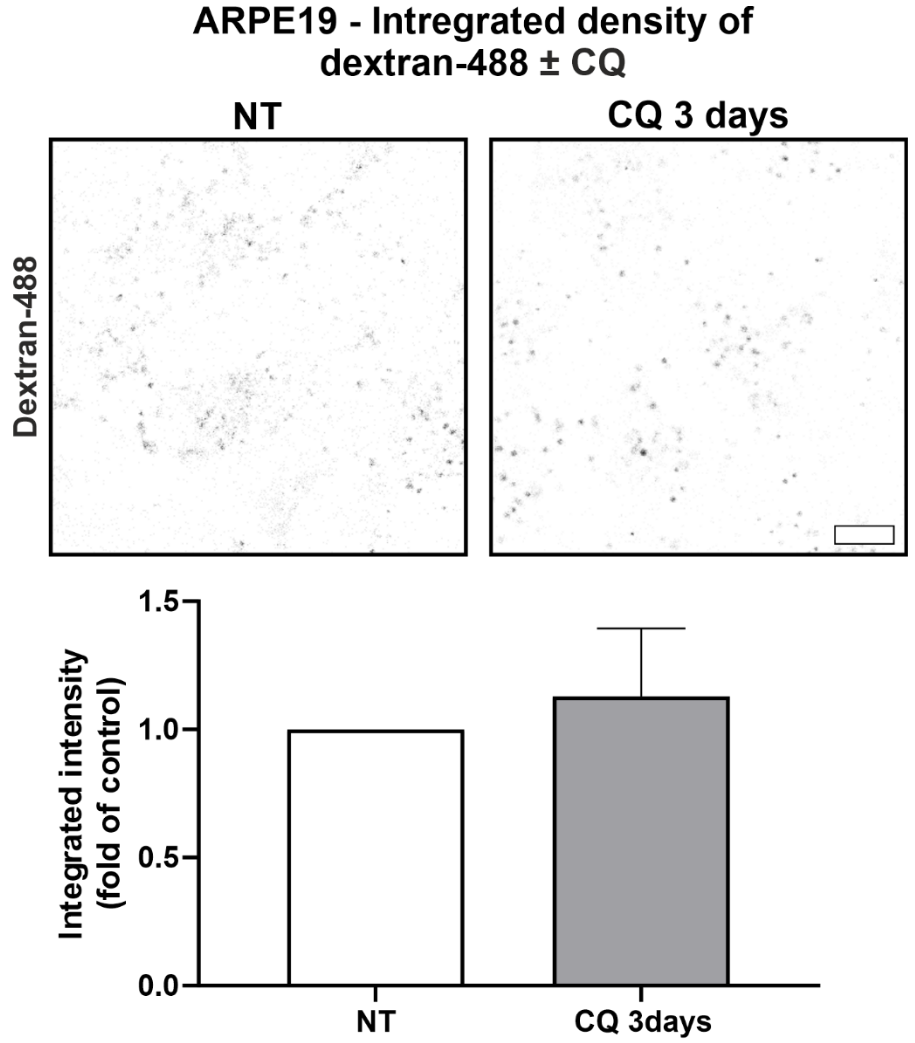

Supplementary Figure 9 Effects of CQ on dextran uptake

The uptake of dextran in ARPE19 was measured from the experiment described in Figure 5, with cells treated with or without CQ for 3 days before a 2 hour pulse of dextran-488. Results are means +/- SEM of 3 independent experiment. No statistical significance was determined by unpaired t-test. Scale bar: 10µm.
